# Cancer Mortality in Louisiana’s Correctional System, 2015-2021

**DOI:** 10.1001/jamanetworkopen.2024.46411

**Published:** 2024-11-20

**Authors:** Totadri Dhimal, Paula Cupertino, Zijing Cheng, Erika E. Ramsdale, Bailey K. Hilty Chu, Brian J. Kaplan, Andrea Armstrong, Xueya Cai, Yue Li, Fergal J. Fleming, Anthony Loria

**Affiliations:** 1 Surgical Health Outcomes & Reaching for Equity, Department of Surgery, University of Rochester Medical Center, Rochester, New York; 2 Department of Public Health Sciences, University of Rochester Medical Center, Rochester, New York; 3 James P. Wilmot Cancer Center, University of Rochester Medical Center, Rochester, New York; 4 Division of Surgical Oncology, Department of Surgery, NYU Grossman School of Medicine, New York, New York; 5 Loyola University New Orleans, College of Law, New Orleans, Louisiana; 6 Department of Biostatistics and Computational Biology, University of Rochester, Rochester, New York; 7 Division of Health Policy and Outcomes Research, Department of Public Health Sciences, University of Rochester Medical Center, Rochester, New York

## Abstract

This cohort study evaluates cancer-specific mortality data about incarcerated individuals in Louisiana to examine why gaps in cause-specific data exist for this group.

## Introduction

Nearly 2 million individuals are incarcerated annually in the US, predominantly low-income men from racial and ethnic minority backgrounds.^1^ The prison population is also aging, with those aged 55 years or older projected to constitute one-third of all incarcerated individuals by 2030.^2^ Imprisonment is associated with accelerated physiological aging, and national data show that incarcerated individuals have 22% higher odds of receiving a cancer diagnosis compared with the general population.^3^ Despite the convergence of multiple cancer risk factors, such as behavioral patterns, barriers to care, and systemic issues, including high prevalence of substance use, HIV, and viral hepatitis in correctional settings, significant gaps persist in understanding cause-specific mortality during incarceration. This study aims to evaluate cancer-specific mortality among incarcerated individuals in Louisiana.

## Methods

This retrospective cohort study received exemption from the University of Rochester Institutional Review Board because it determined that the study did not meet the criteria for human participant research as defined by the Department of Health and Human Services and FDA regulations. The study followed the STROBE reporting guideline. Starting in 2019, Loyola University New Orleans law students collected mortality data about incarcerated individuals through annual public records requests to Louisiana prisons, jails, detention centers, and parish coroners.^4^ Data collection was standardized, transcribed by law students, and verified by 2 physicians for accuracy. Race and ethnicity were obtained and reported from death reports created by the Louisiana Department of Corrections (LADOC). Annual incarcerated population sizes were obtained from the Corrections Statistical Analytics Tool and LADOC, whereas Louisiana’s annual adult population was obtained via the Behavioral Risk Factor Surveillance System. The primary outcome was the age-adjusted cancer-related mortality rate. Age-specific death rates (ASDRs) were computed for each age stratum of male and female patients with cancer using age- and sex-specific standard population weights. 95% CIs were calculated using the Poisson approximation, with 2-sided *P* < .05 being statistically significant. Statistical analysis was performed from January 15 to May 31, 2024, using Microsoft Excel for Microsoft Office 365, version 2406.

## Results

From 2015 to 2021, 24.8% (n = 205) of 828 deaths in LADOC custody were cancer related. Of these, 97.6% (n = 200) were men, and 56.6% (n = 118) identified as Black. Median age at death was 61 years (IQR, 57-66 years) after a median of 12.2 years (IQR, 3.6-24.9 years) in custody. Clinical information was available for 63.4% (n = 130), and specific tumor types were documented for 55.6% (n = 114). Of those with clinical data, 92.3% (n = 120) were evaluated by a physician, 86.9% (n = 113) had undergone diagnostic testing, 86.9% (n = 113) had received medications, 26.9% (n = 35) had surgery, and 93.2% (n = 125) died in a dedicated medical unit. Lung cancer was the most common tumor type, accounting for 36.0% (n = 41), followed by hepatobiliary (15.8% [n = 18]), hematologic (9.6% [n = 11]), pancreatic (8.8% [n = 10]), and colorectal (7.9% [n = 9]) cancers (Table). The cancer-specific ASDR was 158 per 100 000 incarcerated individuals compared with 168 per 100 000 among nonincarcerated Louisiana residents and 149 per 100 000 in the US (Figure). Cancer-related mortality was the highest among those aged 55 years or older (144 vs 4 per 100 000) compared with those who were aged 18 to 54 years.

**Table.** Demographics of Individuals Who Died in Louisiana Department of Correction’s Custody

Variables	Individuals, No. (%)		Individuals living incarcerated, No. (%)^a^
	Cancer-related mortality	Non–cancer-related mortality	
Total No. (%)	205 (24.8)	623 (75.2)	26 377 (100.0)
Age, median (IQR), y	61 (56-67)	60 (52-69)	44
Sex			
Male	200 (97.6)	604 (97.0)	25 137 (95.3)
Female	5 (2.44)	19 (3.05)	1239 (4.69)
Race			
Black	116 (56.6)	373 (59.9)	17 304 (65.6)
White	89 (43.4)	247 (39.7)	8957 (34.0)
Other or unknown^b^	NA	3 (0.4)	116 (0.4)
Duration of incarceration, y			
<5	68 (33.2)	154 (24.7)	15 298 (58.0)
5-9	24 (11.7)	92 (14.8)	4141 (15.7)
10-14	23 (11.2)	68 (10.9)	2347 (8.9)
≥15	88 (42.9)	303 (48.6)	4591 (17.4)
Missing	2 (1.0)	6 (1.0)	NA
Years incarcerated, median (IQR)	12.2 (3.6-24.9)	14.7 (5.0-28.5)	NA
Unspecified preexisting condition	92 (44.9)	236 (37.9)	NA
Location of death			NA
Medical unit	125 (61.0)	394 (63.2)	NA
Housing cell	7 (3.4)	59 (9.5)	NA
Segregation unit	0	3 (0.5)	NA
Other	2 (1.0)	6 (1.0)	NA
Unknown	71 (34.6)	161 (25.8)	NA
Tumor type			
Nonspecific	87 (42.4)	NA	NA
Lung	41 (20.0)	NA	NA
Hepatobiliary	18 (8.8)	NA	NA
Hematologic	11 (5.4)	NA	NA
Pancreatic	10 (4.9)	NA	NA
Colorectal	9 (4.4)	NA	NA
Urologic	9 (4.4)	NA	NA
Esophagogastric	8 (3.9)	NA	NA
Head and neck	5 (2.4)	NA	NA
Breast	1 (0.5)	NA	NA

Abbreviation: NA, not applicable.

^a^ Living incarcerated population as of January 1 to December 31, 2021.

^b^ Other race is not defined in original dataset by the Louisiana Department of Corrections.

**Figure.**
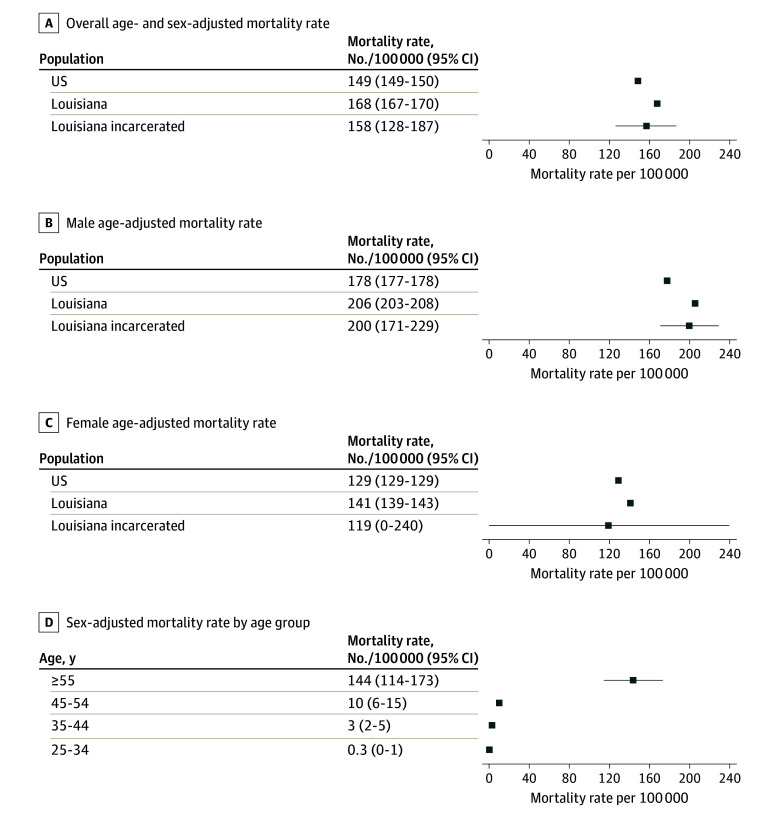
Age- and Sex-Adjusted Cancer-Specific Mortality Rates US indicates the mean US mortality rate.

## Discussion

Human rights, health care disparities, economics, and public policy intersect when evaluating mortality in incarcerated populations. With increasing medical costs, it is critical to evaluate the quality and costs of health care associated with terminally ill incarcerated individuals. Our study found that the cancer-specific mortality rate among incarcerated individuals in Louisiana was higher than the national rate but lower than the state’s, with the majority of these deaths occurring among those older than 55 years.^5^ From 2018 to 2021, the LADOC granted medical release to 72 individuals, whereas 577 individuals with terminal cancer died in custody during the same period. Medical compassionate release remains an underutilized option, and the effect of providing a dignified death for families, health care professionals, incarcerated individuals, payers, and policymakers needs further evaluation.^6^ Limitations of this study include lack of detailed clinical variables, such as treatments offered or received and individual risk factors, which limits the ability to make conclusions about care delivery. Nevertheless, this study highlights the growing burden of cancer diagnosis among incarcerated individuals and underscores the need for rigorous research within this population.

## Notes

Supplement.
Data Sharing Statement
