# Supplementary material for: Cancer Mortality in Louisiana’s Correctional System, 2015-2021
Source: JAMA Netw Open. 2024 Nov 20;7(11):e2446411. doi: 10.1001/jamanetworkopen.2024.46411 (PMC11579789; doi:10.1001/jamanetworkopen.2024.46411)
Supplement: Supplement. — Data Sharing Statement [file jamanetwopen-e2446411-s001.pdf]

Data Sharing Statement

Dhimal. Cancer Mortality in Louisiana's Correctional System, 2015-2021. *JAMA Netw Open*. Published November 20, 2024. doi:10.1001/jamanetworkopen.2024.46411

Data
Data available: No

Additional Information
Explanation for why data not available: Data is publicly available for download.
